# Diversity in high-impact psychiatric publishing: gender parity within reach?

**DOI:** 10.1007/s00737-021-01202-8

**Published:** 2022-01-13

**Authors:** Andrea Gmeiner, Melanie Trimmel, Amy Gaglia-Essletzbichler, Beate Schrank, Stefanie Süßenbacher-Kessler, Michaela Amering

**Affiliations:** 1 Division of Social Psychiatry, Department of Psychiatry and Psychotherapy, Medical University of Vienna, Waehringer Guertel 18-20, 1090 Vienna, Austria; 2 Division of Psychology, Bangor University Wales, Bangor, UK; 3 Department of Adult Psychiatry, Karl Landsteiner University for Health Sciences, University Clinic Tulln, Tulln, Austria

**Keywords:** Academic psychiatry, Gender, Diversity, Authorship

## Abstract

Gender parity and authorship diversity are declared goals in the publishing world. This study assessed the progress of authorship gender distribution over a quarter of a century and geographic diversity over the last 15 years in high-impact psychiatric journals. All articles published in 2019 in the *American Journal of Psychiatry*, the *British Journal of Psychiatry*, and *JAMA Psychiatry* were included and compared with data from three points in time starting in 1994. Descriptive statistics were gathered, and chi-square tests were performed. All tests were conducted as two-tailed, and *p*-values < 0.05 were considered to be statistically significant. Inter-rater reliability was calculated via Cohen’s kappa. In 2019 a total of 473 articles were published. Forty percent of all authors, 42.3% of first authors, and 29.4% of senior authors were female. Counting original research articles only, female first authorship reached 50.4%. In the 25-year period between 1994 and 2019, female first (*p* < .001), female senior (*p* < .001), and female overall (*p* < .001) authorship has increased. In the specific period between 2014 and 2019, overall female senior authorship in all articles (*p* = .940) as well as first (*p* = .101) and senior (*p* = .157) in original research plateaued. In non-original research articles, female first authorship was higher in 2019 compared to 2014 (*p* = .014), whilst female senior authorship plateaued (*p* = .154). Geographic diversity was low and did not change over time. Gender parity in the subcategory original research articles was reached for the first time in 2019. Senior female authorship and geographic diversity remain areas of concern that need further investigation and specific interventions.

## Introduction

Gender parity and authorship diversity are declared goals in the publishing world in academic medicine (Clark and Horton 2019; Upthegrove et al. 2021). Data on gender distribution in authorship in mostly high-impact English-language journals have been published for different medical specialties either focusing on their field (Shah et al. 2021; Thelwall 2020) or on specific journals (Campbell et al. 2019), disorders (Menzel et al. 2019), study designs (Mehran et al. 2021), or publication types (Mamtani et al. 2020). The percentages of female authors in original research articles differ between medical specialties and range from about one-quarter to two-thirds with smaller rates of female first and senior authors in the fields of, e.g., cardiology (Asghar et al. 2018) and anesthesia (Pagel et al. 2019) in contrast to a larger female participation in, e.g., pediatrics (Fishman et al. 2017) and dermatology (Bendels et al. 2018). The overall increasing rates over time relate to female first authorship more than senior authorship. That fact brings to mind the frequently used metaphor of the “leaky pipeline” regarding the career advancement of women in academia and highlights that women are well represented in early career positions but are under-represented at senior levels in the fields of science, technology, engineering, and mathematics (Sheltzer and Smith 2014). There is no indication that academic psychiatry is an exception (Amering et al. 2011; Hart et al. 2019; Süßenbacher et al. 2017).

With respect to the goal of enhancing geographic diversity amongst authors, data from high-impact journals show an overwhelming preponderance of publications from North America and Europe (Filardo et al. 2016).

This study attempts to systematically assess the development of gender distribution of authorship over the last quarter of a century and of geographic diversity over 15 years in high-impact psychiatric journals.

## Methods

A bibliometric review of all articles published during the year of 2019 in three of the most prestigious general psychiatry journals *JAMA Psychiatry* (JP), the *American Journal of Psychiatry* (AJP), and the *British Journal of Psychiatry* (BJP) examined gender distributions in authorship and geographic diversity and compared them with comparable existing data from the years of 1994, 2004, and 2014 for gender and from 2004 and 2014 for geographic diversity. Back in 1994, highest-ranking general psychiatry journals *Archives of General Psychiatry* (now: *JAMA Psychiatry*) and *The American Journal of Psychiatry* as well as the highest-ranking non-American journal *The British Journal of Psychiatry* were chosen due to longstanding consistency in their high-impact factor rank, as well as for ease of comparability to data from previous studies by our group reporting on gender and authorship for the above mentioned years and journals (Amering et al. 2011; Süßenbacher et al. 2017).

Articles listed as published in-print on the journals’ homepages in the year 2019 were included in the analysis. The study group distinguished between original research articles as defined by the journals and non-original research articles such as editorials or letters. Gender was assessed for all authors indicating first and senior authorship with a single author counted as first as well as senior author. Gender was identified by gender-specific given names and by searching university homepages or research gate profiles in cases in which the names of authors did not immediately specify the gender of the author. For the present study, the research team has figured out and discussed the selected categories. Two researchers assessed data from 1 month of each journal separately in order to evaluate the inter-rater reliability; the other 11 months were rated by these two researchers together. Cultural areas and regions according to the United Nations Statistic Division (UNSD, standard country or area codes for statistical use) and income areas (WCP Congress, Country Classification, 2019) were assessed for the first author’s affiliation, because it is widely accepted that the first author is the lead author and the first author’s resources would shape the research described in the article.

### Statistical analyses

The 2019 data were compared to data from the years 1994, 2004, and 2014 (Amering et al. 2011; Süßenbacher et al. 2017). Descriptive statistics (percentages, frequencies) were calculated for each of the three journals. In order to calculate developments over the periods from 1994 to 2004 to 2014 to 2019, chi-square tests were performed. All tests were conducted as two-tailed, and *p*-values < 0.05 were considered to be statistically significant. Inter-rater reliability was calculated via Cohen’s kappa, which was 0.93. All statistical analyses were performed with SPSS (SPSS 28).

## Results

In 2019, a total of 473 articles were published in the *American Journal of Psychiatry* (AJP), the *British Journal of Psychiatry* (BJP), and the *JAMA Psychiatry* (JP) with a total number of 3471 authors listed. One thousand three hundred eighty-six (40%) of all authors listed, 200 (42.3%) of first authors, and 139 (29.4%) of senior authors were female. In the AJP, 498 (38%) of all authors listed, 62 (43.4%) of first authors, and 41 (28.7%) of senior authors were female. In the BJP, 324 (42%) of all authors listed, 46 (34.8%) of first authors and 35 (26.5%) of senior authors were female. In JP, 564 (40.5%) of all authors listed, 92 (46.5%) of first authors, and 63 (31.8%) of senior authors were female. Seventy-three (51%) of the articles in AJP, 66 in BJP (50%), and 100 in JP (50.5%) were assigned to non-original research categories. In the AJP, 101 (70.6%) of first authors were affiliated in the USA, followed by the UK (8.4%) and Canada and the Netherlands (both 5.6%), and 99.3% of first authors were affiliated in high-income countries. In the BJP, 70 (53%) of first authors were affiliated in the UK, followed by Australia (6.8%) and the USA (6.1%), and 90.9% of first authors were affiliated in high-income countries. In JP, 102 (51.5%) of first authors were affiliated in the USA, followed by the UK (12.1%) and Canada (7.1%), and 96% of first authors were affiliated in high-income countries. Results for 2019 are also shown in Table 1.

**Table 1:** Female authorship percentage and overall geographic affiliations in 2019

	Total	BJP	JAMA	AJP
All articles	*N* = 473	*N* = 132	*N* = 198	*N* = 143
Total female authors, *n* (%)	1386 (40)	324 (42)	564 (40.5)	498 (38)
Female first authors, *n* (%)	200 (42.3)	46 (34.8)	92 (46.5)	62 (43.4)
Female senior authors, *n* (%)	139 (29.4)	35 (26.5)	63 (31.8)	41 (28.7)
Original research articles	*N* = 234	*N* = 66	*N* = 98	*N* = 70
Female first authors, *n* (%)	118 (50.4)	30 (45.5)	52 (53.1)	36 (51.4)
Female senior authors, *n* (%)	71 (30.3)	19 (28.8)	30 (30.6)	22 (31.4)
First authors’ affiliations	*N* = 473	*N* = 132	*N* = 198	*N* = 143
High-income countries,* n* (%)	452 (95.6)	120 (90.9)	190 (96)	142 (99.3)
Upper-middle-income countries, *n* (%)	15 (3.2)	8 (6.1)	6 (3)	1 (0.7)
Lower-middle-income countries, *n* (%)	6 (1.3)	4 (3)	2 (1)	-

The total number of publications dropped from 950 in 1994 to 800 in 2004 to 642 in 2014 (Amering et al. 2011; Süßenbacher et al. 2017) and to 473 in 2019.

### Female authorship in all included articles over time

**Fig. 1:**
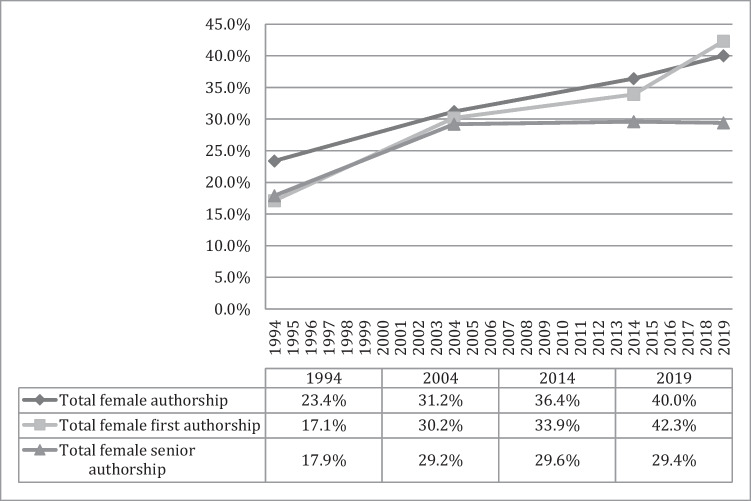
Female authors overall

Between 1994 and 2019, total female authorship in all articles increased significantly (*p* < 0.001); also first authorship (*p* < 0.001) and senior authorship in all included articles increased significantly (*p* < 0.001). Between 2014 and 2019, female authorship in total (*p* < 0.001) as well as first authorship (*p* = 0.005) increased significantly, whilst senior authorship plateaued (*p* = 0.940). Details are shown in Fig. 1.

### Female authorship in original research articles

**Fig. 2:**
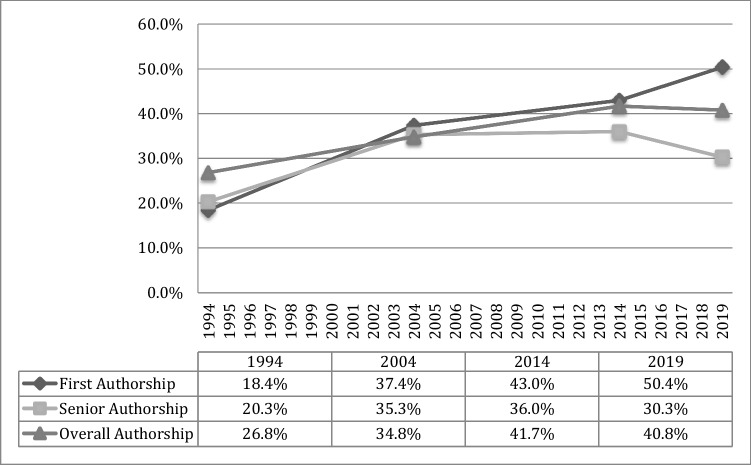
Female authors in original research articles

Focusing on original research articles only, between 1994 and 2019, female first authorship (*p* < 0.001) and senior authorship (*p* = 0.003) increased. First authorship plateaued between 2004 and 2014 (Süßenbacher et al. 2017) and plateaued between 2014 and 2019 (*p* = 0.101) but increased significantly between 2004 and 2019 (*p* < 0.001). Senior authorship has been plateauing between 2004 and 2014 (Süßenbacher et al. 2017) and keeps plateauing between 2014 and 2019 (*p* = 0.157). Details are shown in Fig. 2.

### Female authorship in non-original research articles

**Fig. 3:**
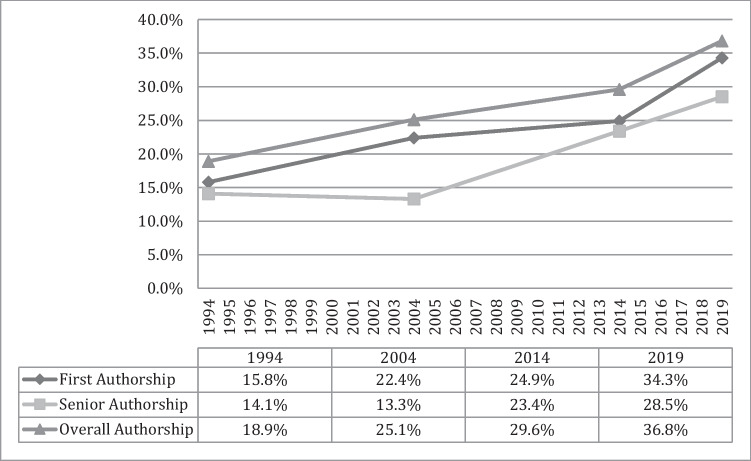
Female authors in non-original research articles

When examining non-original research articles, female first authorship increased significantly between 1994 and 2019 (*p* < 0.001) and 1994 and 2004 (Süßenbacher et al. 2017), plateaued between 2004 and 2014 (Süßenbacher et al. 2017), and increased significantly again between 2014 and 2019 (*p* = 0.014). Female senior authorship increased significantly between 1994 and 2019 (*p* < 0.001) and 2004 and 2014 (Süßenbacher et al. 2017) but plateaued between 2014 and 2019 (*p* = 0.154). Details are shown in Fig. 3.

### Geographic areas

**Fig. 4:**
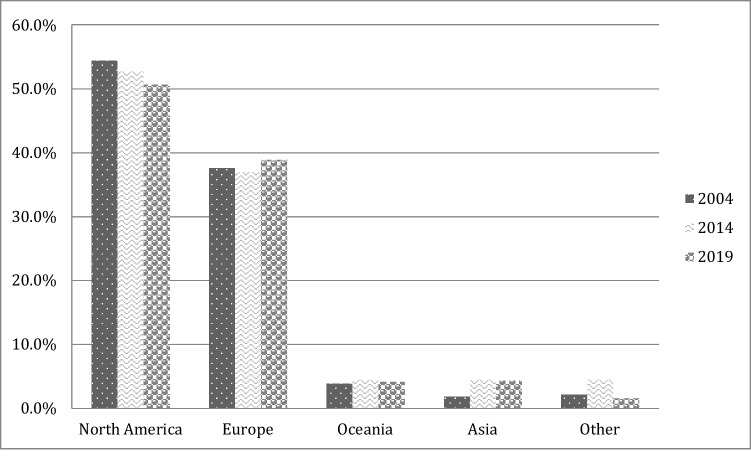
Geographic areas of the 1st authors’ affiliations during the 15-year period in all journals examined

When focusing on all articles published, 95.6% of first authors were affiliated in high-income countries, 3.2% in upper-middle-income countries, and 1.3% in lower-middle-income countries. 50.7% of all articles were published by first authors from North America (USA and Canada) and 38.9% from Europe, a fact that has been stable over the past 15 years (see Fig. 4).

## Discussion and conclusions

The present study explored gender in authorship in three of the highest-impact psychiatric journals over a quarter of a century and geographic diversity over the last 15 years.

Results indicate that gender parity in first authorship was reached in the category of original research articles with a remaining underrepresentation of women in senior positions and showing that most of the first authors were affiliated in high-income countries.

About two-thirds of the first authors were affiliated in the USA or in the UK, and that did not change considerably over the last 15 years. Both of the US-American journals published a majority of articles of US-affiliated authors, whilst the British journal published a majority of articles of UK-affiliated authors. Overall, articles published in the *British Journal of Psychiatry* showed the most geographic diversity with respect to authorship. However, it is a well-known problem that there is a lack of geographic diversity in authoring academic publications (Newton 2020) and the underlying causes are multifactorial.

When making comparisons between different medical disciplines, the current parity of first authorship in original research in psychiatry as well as in pediatrics (Fishman et al. 2017) is in contrast to the disparity in cardiology and anesthesia (Asghar et al. 2018; Pagel et al. 2019), in which women are still under-represented in all categories of authorship. The association of these results with the percentage of women in the respective clinical and academic fields seems an open question that needs further investigation.

The year 2019 is the first year to show gender parity in the important category of original research articles in the three journals studied. These results are similar to findings from a study that examined all published original research articles in 33 high-impact psychiatric journals between 2008 and 2018 (Hart et al. 2019), where results indicated a nearing towards parity in female first authorship, and to Upthegrove et al.’s (2021) recent observation concerning original research articles in the *British Journal of Psychiatry* in the year 2019. Looking at the development over the last quarter of a century, our data show that female first authorship in original research articles has increased significantly since 1994 and since 2004. Overall however, the steep ascent for women as first or senior authors during the nineties and early 2000s (Amering et al. 2011; Süßenbacher et al. 2017) flattened over the last 15 years.

Furthermore, the results of the present study indicate that the number of female senior authors in original research articles has been growing on a lesser rate and stagnating since the early 2000s, bringing the “leaky pipeline” metaphor to mind, which refers to the factor that women are well-represented in early career positions but are under-represented at senior levels (Sheltzer and Smith 2014). Amongst the three journals, the numbers of female authors and the discrepancy between first und senior authors were similar. The *British Journal of Psychiatry* recently highlighted its concern about gender disparity as Upthegrove et al. (2021) provided data on first and senior female authors of primary research papers, reviews, and editorials with gender parity in first authorships and gender disparity for female senior authors in original research articles in the BJP. They did call for “concerted and affirmative action” to *make science more just* and indicated that one of the barriers to correcting these inequalities is the lack of routinely collected data. Discrepancies in development in terms of first and senior authorship were found by Upthegrove et al. (2021), Hart et al. (2019), and by our study group, and that fact needs scientific attention now. Interventions concentrating on the “leaky pipeline” phenomenon are needed.

Apart from possible interventions on a structural level, issues related to the barriers faced by women at an individual level still need to be better understood. During the early time and on the topic of the COVID-19 pandemic, papers published related to that topic showed a majority of male authors overall as well as regarding first and senior author positions (Pinho-Gomes et al. 2020). Furthermore, data report that the number of articles authored by women dropped dramatically during the COVID-19 period (Muric et al. 2021). As in other contexts before, reasons assumed are that women were affected disproportionally by lockdown-related issues such as childcare or homeschooling (Pinho-Gomes et al. 2020).

On a structural level, data indicate that for women, a lack of mentoring and the perception of research activities as lowest work priorities emerge as themes. Journals can address some of the issues related to seniority and leadership within the field through selection of editorial staff that reflect the desired publishing diversity, as literature shows that journals with female editors-in-chief have higher rates of female first authorship (Filardo et al. 2016) and that male reviewers are more likely to accept submissions from male researchers (Clark and Horton 2019). However, it was shown that women are at lower odds of filling these editorial positions and at lower odds of serving as reviewers (Clark and Horton 2019; Lundine et al. 2018), which results in a *circulus vitiosus*. In order to understand that problem more fully, Filardo et al. (2016) suggested that transparency regarding data on, e.g., submissions or the assignments of reviewers and editors of journals would be necessary. A recently published discussion in *The Lancet* pointed to a need for an (Clark and Horton 2019) increased awareness of the underrepresentation of women, of possible glass ceilings and gender bias in, e.g., funding, and recommended reorganization of their internal structures as well as a review of the funding environment.

In the present study, only three journals were examined, and all of them were high-impact journals. In addition, affiliations were examined for first authors only, and no data from low-impact journals were examined. Another limitation is that the study focused on two aspects of diversity, and it is not possible to conclude or discuss changes in any other aspect of diversity such as ethnicity or sexual orientation of authors amongst other topics. Furthermore, a binary gender system according to given names was used, and authors can therefore not state about authors being non-binary or affiliating in any other way besides male and female.

In order to address the question of whether the recent developments represent a plateau, a glass ceiling, and a long-awaited achievement of parity in a core subcategory or an ongoing progress of different speed, regular and explicit monitoring is needed. Besides monitoring, a regular publication of authorship distribution should be provided by academic journals. Furthermore, additional research as well as specific interventions and development of outcome measures should focus on the discrepancies between the rates of first and senior authorship and women in senior academic positions. The hypothesis of the “leaky pipeline” should be a focus of further research attempting to find the leaks and to fix them. With regard to geographic diversity, goals as well as possible interventions would need to be formulated in order to ameliorate that disparity. If geographic diversity is a goal, as pointed out by, e.g., Clark and Horton (2019), specific research on that topic, influencing factors and following intervention studies are needed.

## Data Availability

The dataset used and analyzed during the current study is available on reasonable request.
