# Correction to: Diversity in high‑impact psychiatric publishing: gender parity within reach?

**DOI:** 10.1007/s00737-023-01301-8

**Published:** 2023-02-16

**Authors:** Andrea Gmeiner, Melanie Trimmel, Amy Gaglia‑Essletzbichler, Beate Schrank, Stefanie Süßenbacher‑Kessler, Michaela Amering

**Affiliations:** 1 Division of Social Psychiatry, Department of Psychiatry and Psychotherapy, Medical University of Vienna, Waehringer Guertel, 18‑20, 1090 Vienna, Austria; 2 Division of Psychology, Bangor University Wales, Bangor, UK; 3 Department of Adult Psychiatry, Karl Landsteiner University for Health Sciences, University Clinic Tulln, Tulln, Austria

**Correction to: Archives of Women’s Mental Health (2022) 25:327–333**

**https://doi.org/10.1007/s00737-021-01202-8**

The author noticed that the authors’ surname and given name of the published article entitled *Diversity in high‑impact psychiatric publishing: gender parity within reach?* were presented incorrectly — the surnames and given names of each author were in wrong sequence.

Published version:

Gmeiner Andrea, Trimmel Melanie, Gaglia‑Essletzbichler Amy, Schrank Beate, Süßenbacher‑Kessler Stefanie and Amering Michaela.

Corrected form:

Andrea Gmeiner, Melanie Trimmel, Amy Gaglia‑Essletzbichler, Beate Schrank, Stefanie Süßenbacher‑Kessler and Michaela Amering.

The original article has been corrected.

